# *Mycoplasma suis* Alpha-Enolase Subunit Vaccine Induces an Immune Response in Experimental Animals

**DOI:** 10.3390/vaccines9121506

**Published:** 2021-12-20

**Authors:** Shujiang Xue, Kangseok Seo, Miaosen Yang, Chengdu Cui, Meng Yang, Siyu Xiang, Zongbin Yan, Shengjun Wu, Jincheng Han, Xiaoyang Yu, Yunxiao Li, Xin Jin

**Affiliations:** 1 Department of Veterinary Medicine, College of Agriculture, Yanbian University, Yanji 133002, China; sjxue@ybu.edu.cn (S.X.); cdcui@ybu.edu.cn (C.C.); xiangsiyu1023@163.com (S.X.); 13843945393@163.com (Z.Y.); wushengjunpromab@163.com (S.W.); hjc15904350685@163.com (J.H.); yxyang199719@163.com (X.Y.); 2 Engineering Research Center of North-East Cold Region Beef Cattle Science & Technology Innovation, Ministry of Education, Yanbian University, Yanji 133002, China; 3 Department of Animal Science and Technology, Sunchon National University, Suncheon 57922, Korea; sks@scnu.ac.kr; 4 Department of Chemistry, Northeast Electric Power University, Jilin 132011, China; 20162684@neepu.edu.cn; 5 Department of Pharmacy, Jiangsu Food & Pharmaceutical Science College, Huai’an 223023, China; 20071005@jsfpc.edu.cn; 6 School of Life Science, Shandong University, Qingdao 266237, China

**Keywords:** *Mycoplasma suis*, alpha-enolase, subunit vaccine, immune response

## Abstract

Recombinant protein technology has emerged as an excellent option for vaccine development. However, prior to our study, the immune induction ability of recombinant *Mycoplasma suis* alpha-enolase (rMseno) in animals remained unclear. The purpose of this study was to develop a rMseno protein subunit vaccine and to determine its ability to elicit an immunological response. To accomplish this, we cloned the gene into pET-15b, expressed it in BL21 cells, and purified it. Following the establishment of immunity, the immunogenicity and potential for protection of rMseno were evaluated in mice and piglets. The results demonstrate that anti-*M. suis* serum recognized the pure rMseno protein in both mice and piglets as evidenced by high levels of specific anti-rMseno antibodies, significantly increased levels of IFN-γ and IL-4 cytokines, and significantly increased T lymphocyte proliferation index. Piglets also had significantly increased levels of specific IgG_1_, IgG_2a_, CD4^+^, and CD8^+^ cells. The rMseno findings demonstrated a robust immunological response in mice and piglets, affording partial clinical protective efficacy in piglets.

## 1. Introduction

*Mycoplasma suis* (*M. suis*) is a hemotrophic mycoplasma that has not been successfully cultured in vitro to date [1,2,3,4,5,6]. In pigs, it binds to the surfaces of red blood cells (RBCs), causing erythrocyte malformation and chronic or acute anemia [6,7,8,9,10]. In chronic cases, infected pigs experience emaciation, dysplasia, reproductive dysfunction, and immunosuppression [11,12,13,14]. In acute cases, infected pigs showed high fever, hemolytic anemia, and death in clinical trials. *M. suis* infections have been reported worldwide, including in China, and have resulted in significant economic losses in the swine industry [14,15,16,17]. Some investigations have suggested that humans in close contact with *M. suis*-infected pigs could also become infected, meaning that *M. suis* could jeopardize human health [15,18,19,20]. Recent studies have shown that the immunogenic proteins of *M. suis* are glyceraldehyde-3-phosphate dehydrogenase (GAPDH) protein (previously named MSG1), a DnaK-like heat shock protein (HSPA1), alpha-enolase (ENO1), inorganic pyrophosphatase (PPA), and osialoglycoprotein endopeptidase (OSGEP) [21,22,23,24,25,26,27,28,29,30]. HSPA1 was reported to have an encoding gene that is 1830 bp in size and corresponds to a 67 kDa protein. In experimentally infected pigs, recombinant HspA1 expressed in *Escherichia coli* demonstrated ATPase activity and antigenicity [28]. GAPDH protein was found to be the initial adhesion protein of *M. suis* [22], alpha-enolase was implicated in the adhesion of *M. suis* to porcine red blood cells [21,27], and OSGEP was identified as a membrane surface adhesion protein capable of adhering to host erythrocytes.

Furthermore, it was discovered that OSGEP and GAPDH interact with Band3 and GPA to mediate the adhesion of *M. suis* to porcine erythrocytes [23]. Purified recombinant MSG1, on the other hand, was shown to generate a specific immune response but had no protective effect on the challenged pigs [31].

The recombinant *M. suis* alpha-enolase protein is immunogenic and a promising candidate antigen for developing an anti-*M. suis* vaccine [21]. There is currently a dearth of data on the immunological effect of recombinant alpha-enolase [31]. With this in mind, we isolated recombinant *Mycoplasma suis* alpha-enolase (rMseno) and evaluated its ability to induce an immunological response and protect against infection as a vaccine candidate in piglets. The results indicate that the recombinant *M. suis* alpha-enolase protein induced an immune response in mice and piglets, providing partial clinical protection against *M. suis* challenge in pigs. These clinical data may be utilized to inform the future development of a vaccine to confer resistance to *M. suis* disease.

## 2. Materials and Methods

### 2.1. Design of Experiments

**Figure 1:**
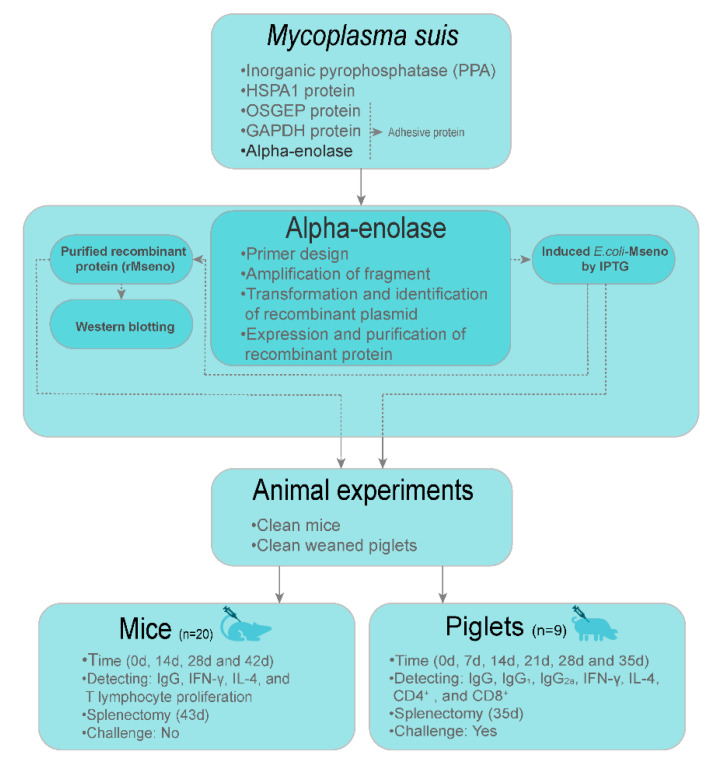
Overall flow.

The workflow is presented in Figure 1. In brief, the first step was to find the target protein; in the second step, we utilized the target protein to develop a recombinant protein vaccine; last, we evaluated immune indices in experimental animals.

### 2.2. Ethics Statement

All animal experiments were conducted in accordance with the Review of Welfare and Ethics of Laboratory Animals guidelines approved by the Jilin Province Administration Office of Laboratory Animals, and the protocol was approved by the Animal Ethics Committee of Yanbian University. All efforts were made to minimize animal suffering.

### 2.3. Cells, Strains, Reagents, and Animals

A total of 20 clean laboratory-grade male Kunming mice aged 4 weeks old and 9 clean weaned piglets aged 40 days old were used in this study. The Laboratory Animal Center of Yanbian University provided the mice, and the piglets were obtained from a farm. The competent *Escherichia coli* BL21 cells were purchased from Sangong Bioengineering (Shanghai, China) and kept by the Preventive Veterinary Laboratory.

Restriction enzymes (*Nde* I and *Xho* I), T4 DNA ligase, and ConA were provided by TaKaRa. The Plasmid Mini I and Gel Extraction kits were provided by OMEGA. The cell proliferation and cytotoxicity detection kit (MIT), protein concentration determination kit (BCA), mouse spleen lymphocyte isolation kit, and porcine spleen lymphocyte isolation kit were obtained from Solarbio. HRP sheep anti-mouse IgG and HRP sheep anti-pig IgG were provided by SIGMA. Mouse anti-*M. suis* serum was prepared by the Preventive Veterinary Laboratory of Yanbian University. The IgG_1_, IgG_2a_, IFN-γ, and IL-4 kits were purchased from Sangon Biotech (Shanghai, China). The RPMI-1640 culture media and fetal bovine serum (FBS) were provided by Biological Industries (Kibbutz Beit-Haemek, Israel).

### 2.4. Primer Design, Synthesis, and Amplification of Target Fragment

The sequence of *Mycoplasma suis* alpha-enolase was optimized according to codon usage bias in an optimization platform provided by Sangon Biotech (Shanghai, China). According to the optimized gene sequence, the primers were designed and synthesized by Sangon Biotech (Shanghai, China). The primer sequences were as follows:

P1: 5′-GACACGACACCATATGGCATTTAGCATTGAAAAC-3′;

P2: 5′-GTGTCCTCGAGTTAGCTTTTAGAGAA-3′.

The target fragment of the alpha-enolase gene was amplified using optimized primers in a 50 μL reaction mixture containing ddH_2_O (38.6 μL), 10× Pfu buffer (5 μL), dNTP (1 μL, 25mM each), P1 and P2 (2 μL each), Pfu (0.4μL), and DNA template (1 μL). The following protocol was utilized for the PCR: pre-denaturalization for 3 min at 95 °C, denaturalization for 22 s at 95 °C, annealing for 20 s at 55 °C, elongation 45 s at 72 °C for a total of 22 cycles, and extension for 5 min at 72 °C. The PCR amplified product (20 μL) was resolved, analyzed in a 1.0% agarose gel electrophoresis, and purified using a QIAquick Gel Extraction Kit.

### 2.5. Construction, Transformation, and Identification of Recombinant pET-15b-ENO Plasmid

PCR products were digested in a 50 μL reaction system (*Nde* I and *Xho* I): purified fragment (20 μL), 10× FD buffer (5 μL), *Nde* I (1 μL), *Xho* I (1 μL), and ddH_2_O (23 μL). The above reaction was performed in a thermostatic water bath for 2 h at 37 °C. The restriction enzyme system of the vector was 50 μL, including pET-15b (1 μg), 10× FD buffer (5 μL), *Nde* I (1 μL), *Xho* I (1 μL), and ddH_2_O (42 μL). The purified target fragment was then linked to the vector. The reaction system (20 μL) consisted of the following components: target fragment digested by enzymes (8 μL), pET-15b digested by enzymes (8 μL), 10× T4 DNA ligase buffer (2 μL), T4 DNA ligase (1 μL), and ddH_2_O (1 μL). The mixture was placed at 22 °C for 1 h. The product was transformed into BL21 cells for 90 s at 42 °C. The positive recombinant plasmid was named pET-15b-ENO. After 2 min on ice, the cells were plated on LB plates and incubated overnight at 37 °C with a final concentration of ampicillin of 50 μg/mL. PCR and restriction digestion (*Nde* I and *Xho* I) were used to extract and evaluate the recombinant plasmid DNA. The BL21 cells, pET-15b vector, and positive recombinant clones were provided by Sangon Bioengineering (Shanghai, China).

### 2.6. Expression, Purification, and Analysis of rMseno

Ten microliters of bacterial broth was inoculated into LB media containing ampicillin (50 μL/mL). We then used IPTG (1 mmol/L) to induce protein expression up to an OD value of 0.6–0.8. After 5 h of incubation, bacteria were collected by centrifugation at 10,000× *g* for 10 min. The bacterial precipitate was combined with 500 μL PBS (pH 7.4) and further broken up by ultrasound for 6 min. We collected the supernatant and precipitation of broken bacteria. In a boiling water bath for 10 min, a 40 μL sample from the precipitation of broken bacteria was dissolved with 500 μL inclusion body solubilization buffer and mixed with 10 μL protein loading buffer. The sodium dodecyl sulfate–polyacrylamide gel electrophoresis (SDS-PAGE) detection system included 12% SDS-PAGE and Tris-Gly buffer subsystems (3.0 g tris, 14.4 g glycine, and 1.0 g SDS in water to make 1000 mL). A 10 μL sample was injected into each electrophoresis lane. The stacking and separating gels were energized for 20 min at 80 v and 60 min at 120 v. The polyacrylamide gel was stained using Coomassie blue staining for 20 min.

The bacterial broth was inoculated in large quantities and broken following the above procedure. The supernatant was collected from broken bacteria and centrifuged for 20 min at 12,000 rpm and 4 °C. Purification of the target protein was accomplished using nickel agarose affinity chromatography. The following procedures were used: To activate the filter function, 5 mL Ni-IDA was soaked in a binding buffer (50 mmol/L Tris, 300 mmol/L NaCl, and pH 8.0). A binding buffer was used to clean the column at a rate of 5 mL per min. Following that, the collected supernatant was injected into the column at a flow rate of 2 mL per min with the target protein. The binding buffer and wash buffer (50 mmol/L Tris, 300 mmol/L NaCl, and 20 or 50 mmol/L imidazolep, pH 8.0) were used to clean the column and remove additional proteins, respectively, at a flow rate of 5 mL per min. The flow rate of the elution buffer (50 mmol/L Tris, 300 mmol/L NaCl, 100 mmol/L or 200 mmol/L imidazole, pH 8.0) was 2 mL per min.

We performed Western blot analyses to probe the purified protein using rat anti-*M. suis* sera as primary antibody (1: 1000). HRP-labeled sheep anti-mouse IgG was used as a secondary antibody (1: 5000). The protein concentration was determined using a BCA kit following the manufacturer’s protocol.

### 2.7. Immunization of Mice

Twenty Kunming mice were randomly assigned into one of four groups, which were immunization groups A and B and control groups C and D. On the first immunization (day 0), each mouse in group A was immunized with 100 μg of pure rMseno protein. Each mouse in group B was inoculated with 2 × 10^7^ CFU-IPTG-induced *E. coli*-Mseno. An equal volume of PBS was used as the control solution for group C, whereas 2 × 10^7^ CFU uninduced recombinant *E. coli*-Mseno was used as the control solution for group D. Injections for the four groups were emulsified with equal volumes of complete Freund’s adjuvant (CFA). For the second and third immunizations (days 14 and 28), each mouse in group A was immunized with 200 μg of pure rMseno protein. Each mouse in group B was inoculated with 4 × 10^7^ CFU-IPTG-induced *E. coli*-Mseno. An equal volume PBS was used for group C, and 4 × 10^7^ CFU uninduced recombinant *E. coli*-Mseno was used for group D. Injections for the four groups were emulsified with equal volumes of incomplete Freund’s adjuvant (IFA). Antibody titers were monitored until day 42. Surgery to remove the spleens of mice was conducted on day 42. All the inoculations were administered via subcutaneous multipoint injections.

### 2.8. Evaluation of Anti-M.-suis Specific Antibody, IFN-γ, and IL-4 in Mouse Serum

Blood samples were collected 0, 14, 28, and 42 days after immunization to determine the concentration of anti-*M.-suis* specific antibodies in the serum using an optimized indirect ELISA [32]. The critical value (cut-off value) for a negative or positive outcome was calculated using the following formula: cut-off = mean value + 3SD, where the mean value was 0.219, the standard deviation (SD) was 0.0185, and the critical value was 0.275. OD_405 nm_ > 0.275 indicated a positive serum sample, while an OD_405 nm_ ≤ 0.275 indicated a negative sample. Mouse serum IFN-γ and IL-4 cytokines were detected according to the manufacturer’s instructions for the IFN-γ and IL-4 ELISA kit.

### 2.9. T-lymphocyte Proliferation Experiment in Mice

On day 42, the mouse spleens were aseptically removed and the lymphocytes were isolated using a mouse spleen lymphocyte isolation kit and cultured for 24 h in vitro. The rMseno, *E. coli*-Mseno, and ConA were used to stimulate the cultured lymphocytes in each group. After 72 h of continuous culture, the proliferation of T lymphocytes was determined using the MTT cell proliferation and cytotoxicity test kit.

### 2.10. Immunization of Piglets

Nine weaned piglets were randomly assigned into three groups of three piglets each. For the initial immunization (day 0), group A received the pure rMseno protein at a dosage of 2.5 mg, whereas group B received 5 × 10^8^ CFU of IPTG-induced *E. coli*-Mseno. Group C received an equal volume of PBS. Injections for the three groups were emulsified with equal volumes of complete CFA. On the second immunization (day 21), group A received the pure rMseno protein at a dosage of 5 mg, whereas group B received 1 × 10^9^ CFU of IPTG-induced *E. coli*-Mseno. Group C received an equal volume of PBS. Injections for the three groups were emulsified with equal volumes of IFA. All the inoculations were administered via subcutaneous multipoint injections.

### 2.11. Evaluation of Anti-M.-suis Specific Antibodies, IFN-γ, and IL-4 in the Porcine Serum

Blood samples were drawn from the jugular vein on days 0, 7, 14, 21, 28, and 35 following immunization. The anti-*M.-suis* specific antibodies in the serum were subsequently determined using an optimized indirect ELISA [32], with optimization conditions consistent with those used for mice. The experiments were performed using the ELISA, IgG_1_, IgG_2a_, IFN-γ, or IL-4 kits.

### 2.12. Detection of T lymphocytes in Piglets

On day 35, the experimental piglets in each group were anesthetized. The spleen was removed surgically, and splenic lymphocytes were isolated using a kit. The cells were then resuspended in RPMI-1640 medium containing 5 % FBS, and Trypan blue staining was performed for counting at a cell dilution of 5 × 10^6^ per mL. Two microliters of fluorescently labeled antibodies was added to 500 μL of diluted cell suspension and incubated in darkness for 30 min. Flow cytometry (FCM) was used to quantify CD4^+^ and CD8^+^ T-cells.

### 2.13. Challenge Experiment

After the second immunization (day 21), the piglets’ spleens were removed on day 35, and 10^9^ high-risk *M. suis* per piglet were subcutaneously injected via multiple points at day 42. Clinical symptoms, body temperature, and bodyweight of the piglets were monitored regularly.

### 2.14. Data Processing

SPSS 17.0 was used for one-way ANOVA analysis among groups, and R 3.6.3 was used for visualization. The *p* value criteria used in the figures are as follows: * *p* < 0.05 presents a significant difference, ** *p* < 0.01 shows a highly significant difference, and *** *p* < 0.001 shows an extremely significant difference.

## 3. Results

### 3.1. Target Gene Amplification and Analysis 

The *M. suis* alpha-enolase gene was successfully amplified at a length of 1632 bp, an increase of 9 bp over the initial length before optimization. After the recombinant positive plasmid pET-15b-ENO was amplified by PCR, we performed SDS-PAGE. A protein band was present at 1632 bp. After *Nde* I and *Xho* I digested the recombinant plasmid pET-15b-ENO, we performed SDS-PAGE. Two protein bands were present at 5700 bp and 1632 bp. The observed values were consistent with the predicted theoretical values.

### 3.2. Expression, Purification, and Identification of Recombinant Protein

**Figure 2:**
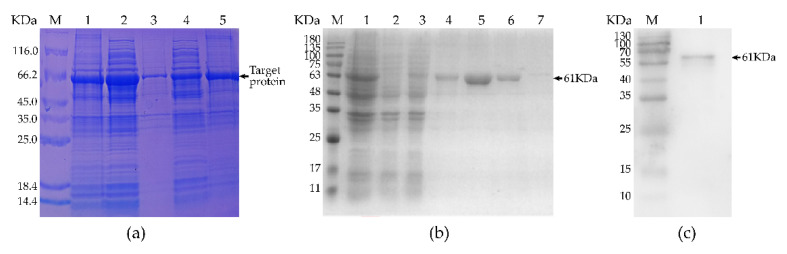
Western blotting to detect the purified recombinant protein. (**a**) M indicates the protein standard marker. The first lane presents the total protein before induction. Lanes 2 to 5 indicate the supernatant lysate of induced recombinant bacteria at 20 °C, the precipitate of the lysate of induced recombinant bacteria at 20 °C, the supernatant lysate of induced recombinant bacteria at 37 °C, and precipitate of the lysate of induced recombinant bacteria at 37 °C, respectively. (**b**) M indicates the protein standard marker. The first lane shows the total protein before induction. The second lane contains the filtered fluid containing the residual protein (excluding the target protein) from the supernatant of lysate of induced recombinant bacteria. The supernatant of the lysate of induced recombinant bacteria containing both bacterial proteins and the target protein is presented in the third lane. The fourth lane is target protein purified with elution buffer containing 100 mmol/L imidazole. The fifth lane is target protein purified with elution buffer containing 200 mmol/L imidazole. The wash buffer elution (50 mmol/L imidazole) is shown in the sixth lane (target protein and hybrid protein). The wash buffer elution containing 20 mmol/L imidazole is presented in the seventh lane (target protein and hybrid protein). (**c**) M indicates the standard protein marker. The first lane shows the purified rMseno protein.

We isolated total bacterial protein before induction, supernatant lysate of induced recombinant bacteria at 20 or 37 °C, and precipitate of the lysate of induced recombinant bacteria at 20 or 37 °C and separated them by electrophoresis. Figure 2a shows that the recombinant protein expression from the induced expression of recombinant bacteria at 37 °C was higher than that at 20 °C. Since both the supernatant and precipitate included the recombinant protein, it existed both dissolved in solution and in inclusion bodies. To facilitate subsequent experimentation, we used recombinant protein isolated from the supernatant of the recombinant bacteria lysate.

As shown in Figure 2b, SDS-PAGE analysis was used to determine the total bacterial protein prior to induction. For filtering with a purification column, we used the supernatant of the lysate of induced recombinant bacteria, elution buffer containing 100 mmol/L or 200 mmol/L imidazoles, and wash buffer containing 20 or 50 mmol/L imidazoles. The results suggest that the treatment for the fifth lane had the greatest purifying impact. The Western blot analysis shown in Figure 2c revealed a band of the recombinant protein at the expected size and demonstrated that the purified recombinant fusion protein had good immunogenicity.

### 3.3. Detection of Anti-M. suis-Specific Antibodies in the Sera of Immunized Mice

**Figure 3:**
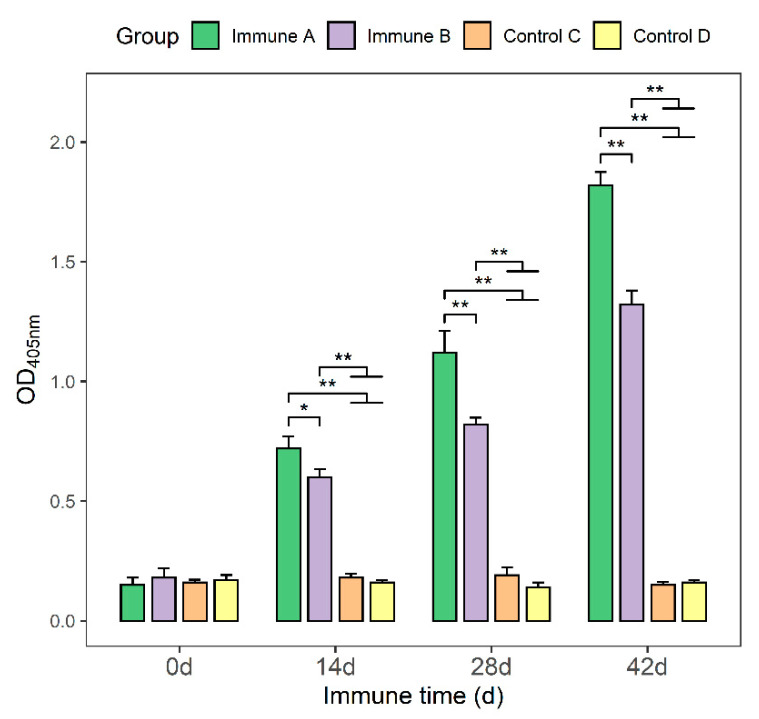
Antibody titers in sera of immunized mice. The OD_405 nm_ was calculated for each group of mice (*n* = 5). An error bar shows the standard error of the mean. Mice in group A received the purified rMseno protein. Mice in group B received IPTG-induced *E. coli*-Mseno. Control group C received an equal volume of PBS. Control group D received uninduced recombinant *E. coli*-Mseno. Each group received three immunizations on day 0, day 14, and day 28. Antibody titers were determined until day 42. * *p* < 0.05 presents a significant difference, and ** *p* < 0.01 shows a highly significant difference.

The protein concentrations in serum samples were determined using an optimized indirect ELISA technique. Following the initial immunization and subsequent booster immunization in groups A and group B, an increase in specific antibodies against the *M. suis* protein in the mouse serum was detected (Figure 3). In comparison, groups C and D were unable to produce specific antibodies. On days 14, 28, and 42, the antibody titers in groups A and B were significantly higher than those in groups C and D (*p* < 0.01). Moreover, the antibody titers in group A were significantly higher than those in group B (*p* < 0.05, *p* < 0.01).

### 3.4. Detection of IFN-γ and IL-4 Cytokines in the Sera of Immunized Mice

**Figure 4:**
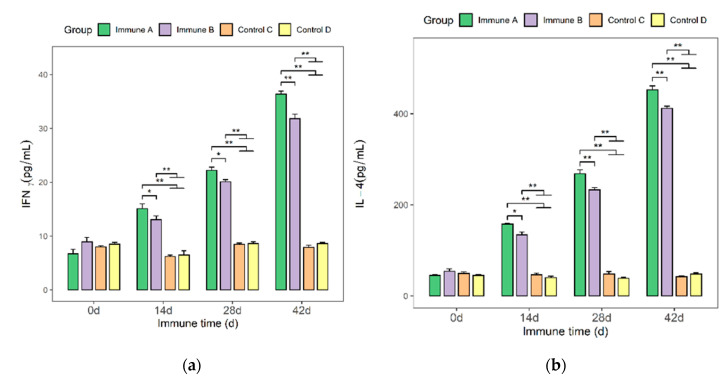
Serum IFN-γ and IL-4 levels in mice from different immunization groups. Each mouse in each group (*n* = 5 per group) was screened for IFN-γ and IL-4. Statistical comparisons were made between groups at all stages of immunization. (**a**) On day 14, IFN-γ levels in immune groups A and B were significantly increased (*p* < 0.05). (**b**) On day 14, the levels of IL-4 in immune groups A and B were significantly increased (*p* < 0.05). * *p* < 0.05 presents a significant difference, and ** *p* < 0.01 shows a highly significant difference.

The levels of IFN-γ and IL-4 cytokines in the sera of immunized mice were determined using an ELISA kit (Figure 4a,b). On days 14, 28, and 42, the levels of IFN-γ and IL-4 were significantly higher in the immunized groups (A and B) than in the two control groups (*p* < 0.01). Similarly, group A had significantly higher cytokine levels than group B (*p* < 0.05 or *p* < 0.01).

### 3.5. T-Lymphocyte Proliferation in Immunized Mice

**Figure 5:**
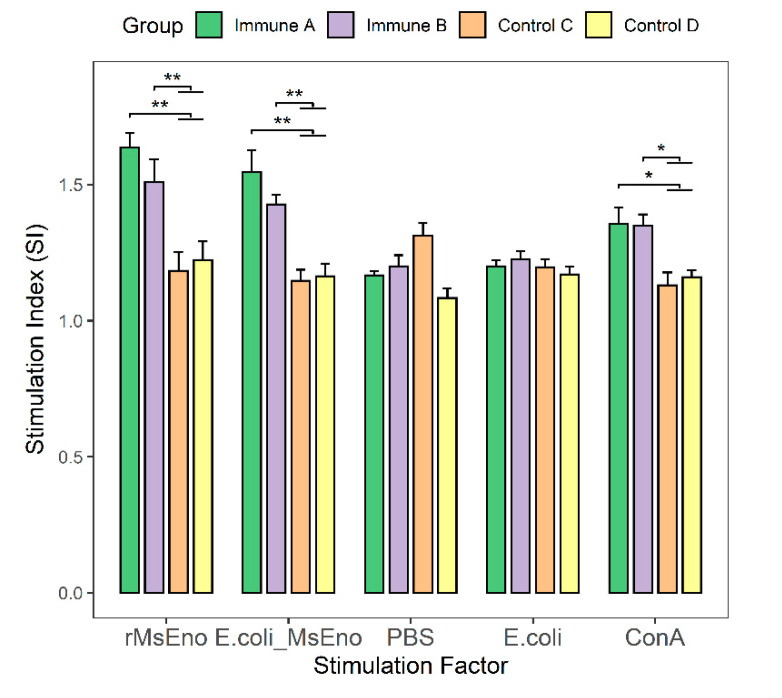
The proliferation of T lymphocytes in mice. The mice were divided into four groups (*n* = 5 per group) and stimulated with rMseno, *E. coli*-Mseno, PBS, *E. coli*, and ConA, respectively. Between groups, statistical comparisons were made using different stimulating factors. * *p* < 0.05 presents a significant difference, and ** *p* < 0.01 shows a highly significant difference.

With the exception of groups that received PBS and *E. coli*, there were highly significant differences between immunization and control groups. The T-lymphocyte proliferation indexes of immunological groups A and B were significantly higher than those of the two control groups when stimulated with rMseno or *E. coli*-Mseno (*p* < 0.01) (Figure 5). Proliferation was significantly greater in groups A and B than in the control groups in response to nonspecific stimulation with ConA protein (*p* < 0.05).

### 3.6. Detection of IgG, IgG_1_, and IgG_2a_ Antibodies in Immunized Piglets

**Figure 6:**
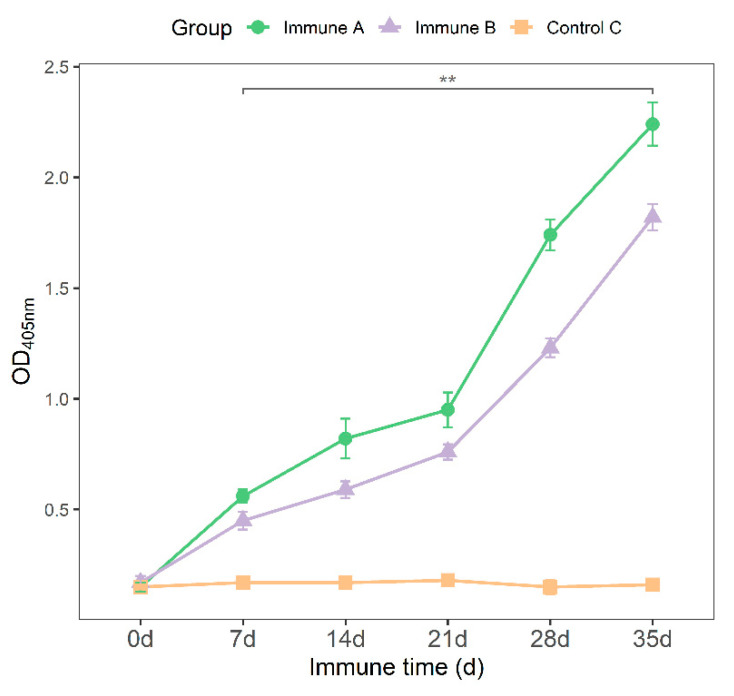
Antibody levels in the sera of piglets. The case groups were divided into groups A and B (*n* = 3 per group) immunized with rMseno and *E. coli*-Mseno, respectively. The control group (*n* = 3 per group) received an equal volume of PBS (C). Two stars above the black line indicate that immune groups A and B were highly significantly different compared to control group C at all points in time (7, 14, 21, 28, and 35 d). ** *p* < 0.01 shows a highly significant difference.

All groups received their first and second immunizations on days 0 and 21, respectively. On days 0, 7, 14, 21, 28, and 35, blood samples and antibodies were obtained and analyzed. The optimized indirect ELISA assay was used to determine the levels of IgG at each time point. The results show that serum IgG antibodies in groups A and B increased gradually following the first vaccine and then increased sharply following the second immunization, a phenomenon not observed in the control groups (Figure 6). The variance analysis revealed that from day 7, the serum IgG antibody levels of piglets in groups A and B were significantly higher than those of group C piglets (*p* < 0.01).

**Figure 7:**
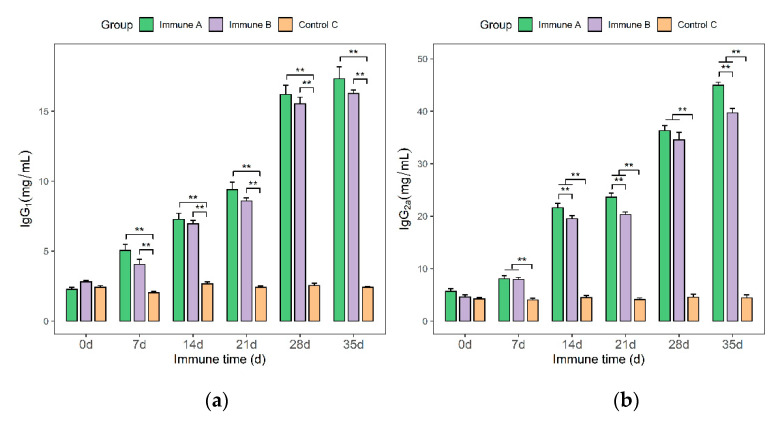
Serum IgG_1_ and IgG_2a_ levels of piglets in the different immunization groups. (**a**) From day 7, the differences between groups A and B (vaccinated) and group C (control) were highly significant, and the IgG_1_ level gradually increased over time in the former two groups. (**b**) From day 7, the differences between groups A and B and group C were highly significant, and the IgG_2a_ gradually increased over time in the former two groups. ** *p* < 0.01 shows a highly significant difference.

As shown in Figure 7, IgG_1_ and IgG_2a_ antibodies were detected in the sera of piglets in each group using IgG_1_ and IgG_2a_ kits. After day 7, the serum IgG_1_ and IgG_2a_ levels of piglets in groups A and B were significantly higher than those in the control group (*p* < 0.01). However, the IgG_1_ levels in immune group A did not differ significantly from the IgG_1_ levels in immune group B at any point in time (7, 14, 21, 28, and 35 days). On days 14, 21, and 35, IgG_2a_ from immunological group A was significantly different from IgG_2a_ from immune group B (*p* < 0.01). The amounts of IgG_1_ and IgG_2a_ antibodies increased continuously in groups A and B (Figure 7a,b). Thus, the data demonstrated that rMseno and *E. coli*-Mseno were immunogenic and capable of eliciting strong humoral immune responses.

### 3.7. Detection of IFN-γ and IL-4 Cytokines in the Sera of Immunized Piglets

**Figure 8:**
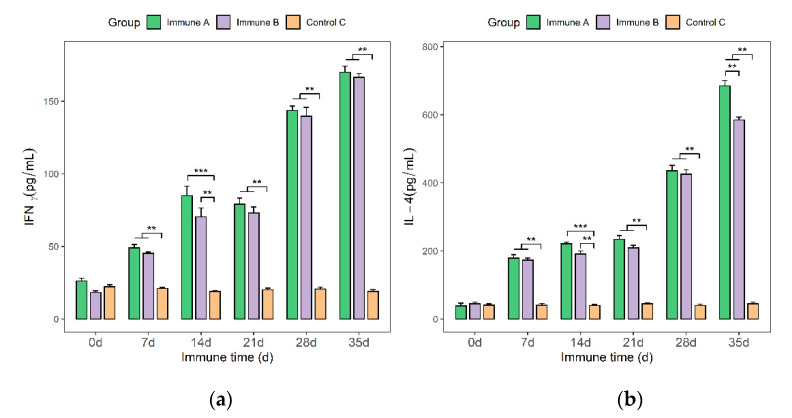
Serum IFN-γ and IL-4 levels in piglets from various immunization groups. (**a**) After the first immunization, the level of IFN-γ in group A was highly significantly different from that of group C (*p* < 0.01 or *p* < 0.001). (**b**) After the initial vaccination, the level of IL-4 in group A was highly significantly different than that of group C (*p* < 0.01 or *p* < 0.001). ** *p* < 0.01 shows a highly significant difference, and *** *p* < 0.001 shows an extremely significant difference.

On days 0, 7, 14, 21, 28, and 35, blood samples were taken from the jugular veins of piglets. IFN-γ and IL-4 cytokines were quantified using an ELISA kit. Within two weeks of the first immunization, the IFN-γ assay findings indicated an increasing trend in the levels of IFN-γ in groups A and B, followed by a slowly decreasing trend that began to rise again after the second immunization. As expected, IFN-γ levels were nearly constant in the control group. Additionally, analysis of variance revealed that the serum IFN-γ levels of piglets in groups A and B were significantly higher than those of piglets in group C (*p* < 0.01 or *p* < 0.001). Piglets in group A had higher serum IFN-γ levels than piglets in group B (Figure 8a). There was, however, no statistically significant difference between the two groups.

Additionally, the results of IL-4 levels in piglet sera demonstrated a tendency of gradual increase following the first vaccine, followed by a significant spike following the second immunization. Within the two weeks following the initial immunization, the IL-4 contents in groups A and B showed slowly increasing trends after the first immunization, followed by moderately increasing trends. Upon the second immunization, the IL-4 levels in groups A and B showed gradual increasing trends, followed by sharper increasing trends. After the second vaccine, the IL-4 levels increased and remained elevated. In the control group, C, the level of IL-4 remained nearly unchanged.

The variance analysis for each experimental group showed that the serum IL-4 levels of piglets in groups A or B were highly significantly or extremely significantly different from those of group C (*p* < 0.01 or *p* < 0.001). From day 7, group A had a slightly higher IL-4 concentration than those of group C and group B. Additionally, on day 35, there was a significant difference between groups A and B. As mentioned previously, the results suggest that rMseno protein and *E. coli*-Mseno may induce superior immune protection.

### 3.8. Evaluation of CD4^+^ and CD8^+^ T-cell Levels in the Immunized Piglets

**Figure 9:**
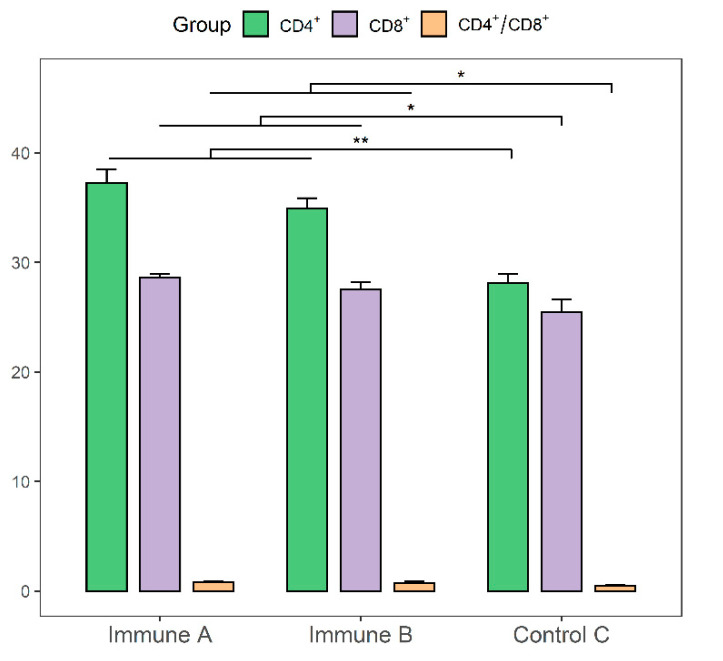
T lymphocytes were analyzed in the spleens of piglets from different immunization groups. The CD4^+^ and CD8^+^ levels were evaluated on day 35. * *p* < 0.05 presents a significant difference, and ** *p* < 0.01 shows a highly significant difference.

CD4^+^ and CD8^+^ T-cell levels in the spleen lymphocytes of immunized piglets were detected and analyzed using flow cytometry, as shown in Figure 9. The CD4^+^ counts in groups A and B were significantly higher than those in group C (*p* < 0.01). CD8^+^ T cells in groups A and B were significantly more numerous than in group C (*p* < 0.05). The ratios of CD4^+^ to CD8^+^ cells in groups A and B were significantly higher than that of group C (*p* < 0.05). These results demonstrated that both rMseno and *E. coli*-Mseno stimulated the production of a strong cellular immune response.

### 3.9. The Outcome of Experimental Challenge

On day 42, the challenge experiment was performed. There were no significant differences in clinical symptoms, body temperature, or body weight between the case and control groups after 1 week. However, the pathogen was first detected 48 h after the challenge in three piglets in group A and for the first time in one piglet, two piglets, and three piglets in group B after 48, 24, and 24 h in group C. The results of the challenge to the piglets are shown in Supplementary Table S1.

### 3.10. The Alpha-Enolase (ENO1) Protein Interaction Network

**Figure 10:**
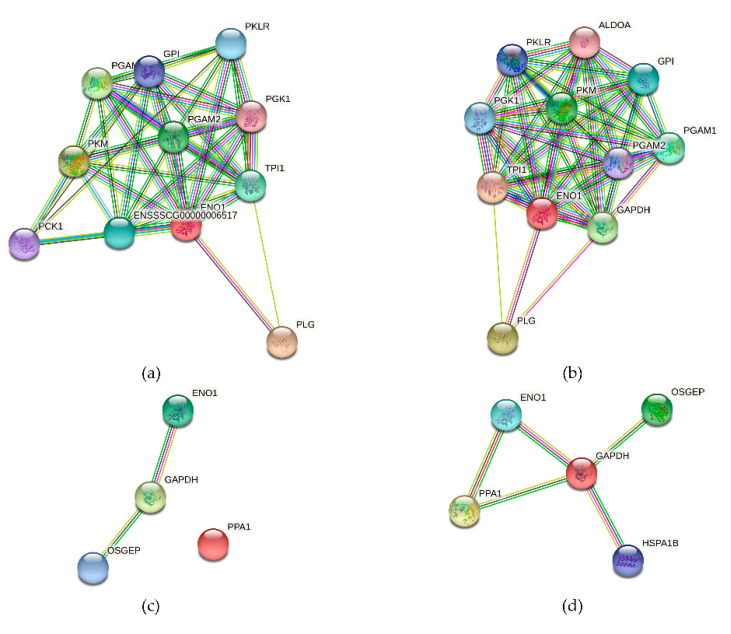
The networks of interactions among alpha-enolase (ENO1) and other proteins (no clustering). Each node represents all the proteins produced by a single, protein-coding gene locus. An association means that the proteins most likely collaborate to perform a common function. The dots and lines are defined in the STRING manual. (**a**) An alpha-enolase interaction network based on *Sus scrofa*. (**b**) An alpha-enolase interaction network based on *Homo sapiens*. (**c**) An interaction network for alpha-enolase (ENO1), GAPDH, and OSGEP in *Sus scrofa*. (**d**) An interaction network for alpha-enolase (ENO1), GAPDH, OSGEP, PPA1, and HSPA1 in *Homo sapiens*.

We constructed an ENO1-centric network based on the protein data from *Sus scrofa* and *Homo sapiens* in the STRING 11.5 database. In the integrated networks (Figure 10a,b), a considerable number of overlapping genes appear around alpha-enolase, and the networks have a high degree of similarity. This implies that these stable and shared functional networks associated with alpha-enolase are consistent in both species. Alpha-enolase, GAPDH, and OSGEP share a stable network, implying a co-expressive relationship between alpha-enolase and GAPDH (Figure 10c,d). Thus, we hypothesized that OSGEP is another important potential vaccination target for *M. suis* and is involved in a network associated with alpha-enolase. Although PPA1 lacks interaction with alpha-enolase in Figure 10c, alpha-enolase strongly interacts with GAPDH and PPA1 in Figure 10d. This suggests an intriguing interaction among these proteins associated with *M. suis* that warrants further investigation.

## 4. Discussion

*Mycoplasma suis* (*M. suis*), is a member of the Mycoplasmataceae family and is thought to be one of only a few *Mycoplasma* species capable of cell invasion [33]. Further studies have shown that it is a member of the uncultivable hemotrophic mycoplasmas, and it has primarily been identified as a surface parasite on in porcine red blood cells [5,14,27,34]. The pathogen is capable of destroying red blood cells directly by adhesion, invasion, nutrient scavenging, immune-mediated lysis, endothelial targeting, and structural changes [1,15,33]. Therefore, adhesion to red blood cells is a critical phase in the unique lifecycle of *M. suis* [27]. Due to the unique RBC-dependent lifestyle of *M. suis*, the identification of adhesive proteins and their interactions on red blood cells is highly critical and significant [6,23,27]. In this study, scanning electron microscopy (SEM) and transmission electron microscopy (TEM) were used to observe the morphological features of high-risk *M. suis*, as shown in Supplementary Figure S2. These findings confirmed the interactions between *M. suis* and RBCs—consistently with previous studies—such as the adhesion and invasion (Supplementary Figure S2d,f) [1,33,35]. Furthermore, mature *M. suis* were used to demonstrate the process of reproduction via splitting (Supplementary Figure S2e). Such continuous adhesion and invasion can cause the most typical severe icteroanemia and other pathological features (Supplementary Figures S1 and S2a,b). These clinical manifestations were observed in a case from which we collected high-risk strains in our neighborhood. The manifestations were consistent with those in a previous study [5].

To date, the identification of adhesion structures has been difficult. Due to the lack of a culture system, only a few adhesion proteins have been identified, including alpha-enolase, GAPDH, and the OSGEP protein. Previously, it was demonstrated that the *M. suis* alpha-enolase could be employed as a complementary antigen to improve the specificity and sensitivity of the serological diagnosis of *M. suis* infections [21]. Additionally, the immunogenicity and adhesive properties of recombinant alpha-enolase produced in *Escherichia coli* were demonstrated in experimentally infected pigs [21]. Our previous research focused on the identification of alpha-enolase and the screening of likely candidate proteins that interact specifically with *M. suis* target proteins. These findings suggested that it was maybe the second most critical adhesion protein after GAPDH [27]. It has previously been demonstrated that alpha-enolase interacts with other proteins in the cytoplasm and with proteins on the porcine RBC membrane (due to its surface accessibility on the *M. suis* cell surface) [21]. Notably, those findings are consistent with our findings for the interaction network (Figure 10). Alpha-enolase is not only an adhesive protein, but is also likely involved in other roles concerning glycolysis [6,36]. The results of the protein interaction network are worthy of further study. In brief, alpha-enolase is considered a vaccine candidate with potential research value.

Antibodies specific for *M. suis* significantly suppress infection throughout the early stages of infection, serving as a primary indicator of vaccine-induced humoral immunity [29,31,37]. In this study, we immunized mice and piglets with rMseno and *E. coli*-Mseno to evaluate the protein’s ability to induce an immune response. An optimized indirect ELISA was then used to determine the levels of specific IgG antibodies in serum. The results showed that specific antibodies against *M. suis* in the two immunization groups were significantly elevated compared to those in the two control groups, indicating that the protein can elicit a more favorable humoral immune response.

In addition, Th2 cells mainly secrete IL-4 and IL-5 cytokines, and the primary function of IL-4 is to promote the proliferation and differentiation of B lymphocytes into plasma cells, inducing an increase in specific IgE IgG_1_-type antibodies to mediate humoral immunity [38,39,40]. IFN-γ is a signature Th1 cytokine that can activate antigen-presenting cells, promote the differentiation of Th1 cells by upregulating the transcription factor T-bet, and induce the production of IgG_2a_ antibodies to enhance the recognition stage of the immune response [41,42]. These findings demonstrate how cytokines can alter the intensity of an immune response and the type of immunological response. Therefore, monitoring changes in cytokine levels can aid in determining the direction and strength of the immune response following vaccination [31]. In this study, the cytokines IL-4 and IFN-γ were used to evaluate the efficacy of vaccines. These findings provide an excellent explanation for the trend that we noticed in the immunological response.

Additionally, the T-lymphocyte proliferation experiment is a method used to detect the functions of T lymphocytes in vitro and the antigen-specific proliferation of T lymphocytes in the body [31,40]. When T cells are grown in vitro, they can be transformed from small lymphocytes into lymphocytes via stimulation with specific antigens or mitogen-promoting agents [31,40]. Their conversion rate reflects cellular immunity levels in the body [31,40]. We investigated the expression levels of IL-4 and IFN-γ, and the proliferation index of the T lymphocytes in the immune group, both of which were significantly higher compared to those of the control group, indicating that the recombinant protein could induce a better cellular immune response in mice and piglets.

The challenge experiment demonstrated that the recombinant protein vaccine provides limited clinical protection against rMseno. Moreover, the results indicated that the partial protection induced a flaw in that the time interval between challenge and pathogen detection was extended by 24 h compared to unimmunized pigs.

In conclusion, this study demonstrated that rMseno elicited good, persistent antibody titers in all immunized mice and piglets, which is necessary. However, neutralizing antibodies and T-cell reactions were not enough to support complete protection. Vulnerable piglets were provided only partial protection against *M. suis*. Subsequent work will focus on improving rMseno-induced neutralizing antibodies and specific T-cell responses—for instance, using molecular adjuvants and prime–boost immune strategies. Further research may be conducted to determine the associations of the interacting proteins with alpha-enolase and to provide additional evidence for the development of a highly effective vaccine for *M. suis*.

## Data Availability

All data supporting the findings of this study are included within the article or supplementary material.

## Supplementary Materials

The following are available online at https://www.mdpi.com/article/10.3390/vaccines9121506/s1. Figure S1. The main model symptom and the pathological change of high-risk M.suis in animal clinical, Figure S2. The morphological features of high-risk M.suis under microscopic state from infected pigs, Figure S3. Expression, purification of recombinant protein, and full-length blots in parallel experiments. Table S1. The results after challenge experients of piglets.
