# Supplementary material for: Mycoplasma suis Alpha-Enolase Subunit Vaccine Induces an Immune Response in Experimental Animals
Source: Vaccines (Basel). 2021 Dec 20;9(12):1506. doi: 10.3390/vaccines9121506 (PMC8708218; doi:10.3390/vaccines9121506)
Supplement: Supplementary file 1 [file vaccines-09-01506-s001.zip › vaccines-1415229-supplementary.pdf]

Mycoplasma suis Alpha-Enolase Subunit Vaccine Induces an Immune Response in Experimental Animals

Shujiang Xue [1,2], Kangseok Seo [3], Miaosen Yang [4], Chengdu Cui [1], Meng Yang [5], Siyu Xiang [1], Zongbin Yan [1], Shengjun Wu [1], Jincheng Han [1], Xiaoyang Yu [1], Yunxiao Li [6,*] and Xin Jin [1,2,*]

[1] Department of Veterinary Medicine, College of Agriculture, Yanbian University, Yanji, China, 133002; sjxue@ybu.edu.cn (S.X.); cdcui@ybu.edu.cn (C.C.); xiangsiyu1023@163.com (S.Y.X.); 13843945393@163.com (Z.Y.); wushengjunpromab@163.com (S.W.); hjc15904350685@163.com (J.H.); Yxyang199719@163.com (X.Y.); jinxin@ybu.edu.cn (X.J.)

2 Engineering Research Center of North-East Cold Region Beef Cattle Science & Technology Innovation, Ministry of Education, Yanbian University, Yanji, China, 133002

3 Department of Animal Science and Technology, Sunchon National University, Suncheon 57922, South Korea; sks@scnu.ac.kr

4 Department of Chemistry, Northeast Electric Power University, Jilin, China, 132011; 20162684@neepu.edu.cn

5 Department of Pharmacy, Jiangsu Food&pharmaceutical Science College, Huaian, China, 223023; 20071005@jsfpc.edu.cn

6 School of Life Science, Shandong University, Qingdao, China, 266237; yunxiao.li@sdu.edu.cn (Y.L.)

* Correspondence: yunxiao.li@sdu.edu.cn (Y.L.); jinxin@ybu.edu.cn (X.J.); Tel.: +86-0433-243-5550 (X.J.)

Supplementary information

Supplementary Figure S1. The main model symptom and the pathological change of high-risk *M.suis* in animal clinical.

Supplementary Figure S2. The morphological features of high-risk *M.suis* under microscopic state from infected pigs.

Supplementary Figure S3. Expression, purification of recombinant protein, and full-length blots in parallel experiments.

Supplementary Table S1. The results after challenge experients of piglets.

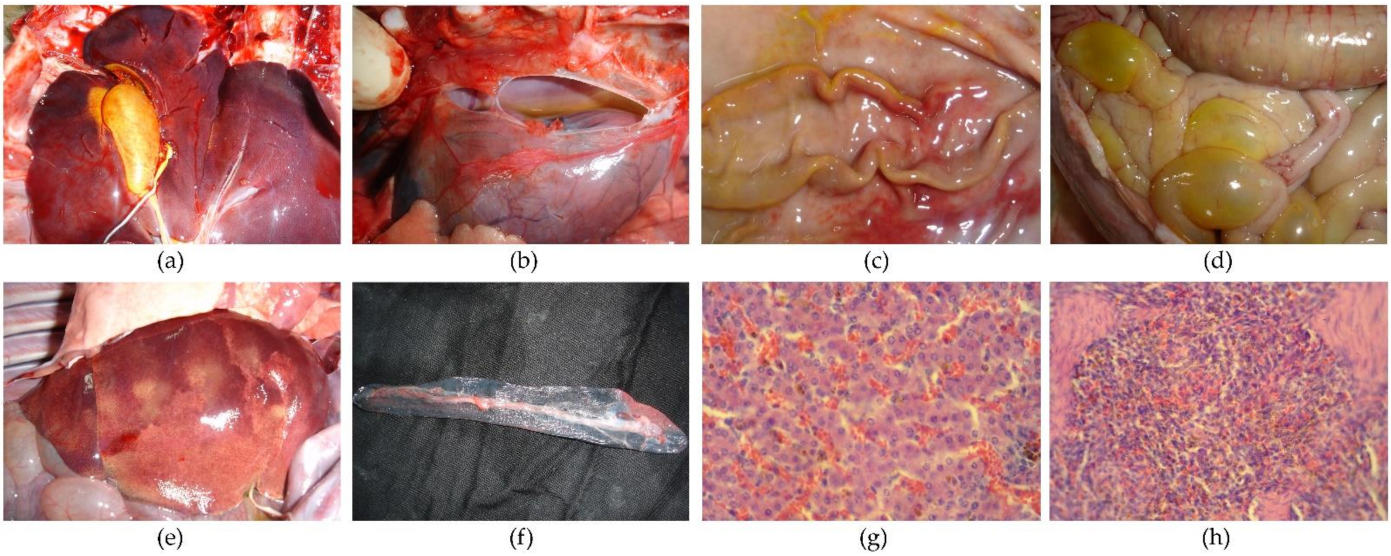

Supplementary Figure S1. The main model symptom and the pathological change of high-risk *M.suis* in animal clinical. In Figure S1g and S1h, HE-strained hepatic and splenic tissue smears from infected pigs were observed under optical microscope. (a) to (d) The color yellow staining caused by *M.suis* was shown in the gallbladder, epicardial fat, gastric mucosa, and small intestine, respectively. (e) The liver was congested and swollen, presented wheat on the surface. (f) The spleen was soft and brittle, presented dark purple on the surface. (g) Some liver cells were necrotic and disintegrated. The hepatic sinusoids were dilated, and that a number of the red cells were seen in it. A small number of cells in the cytoplasm containing hemosiderin were distributed in hepatic sinusoids; Magnification, ×400. (h) The spleen presented bleeding, and numerous iron-swallowing cells were seen in it. Extensive lymphocytes were degenerate; Magnification, ×400.

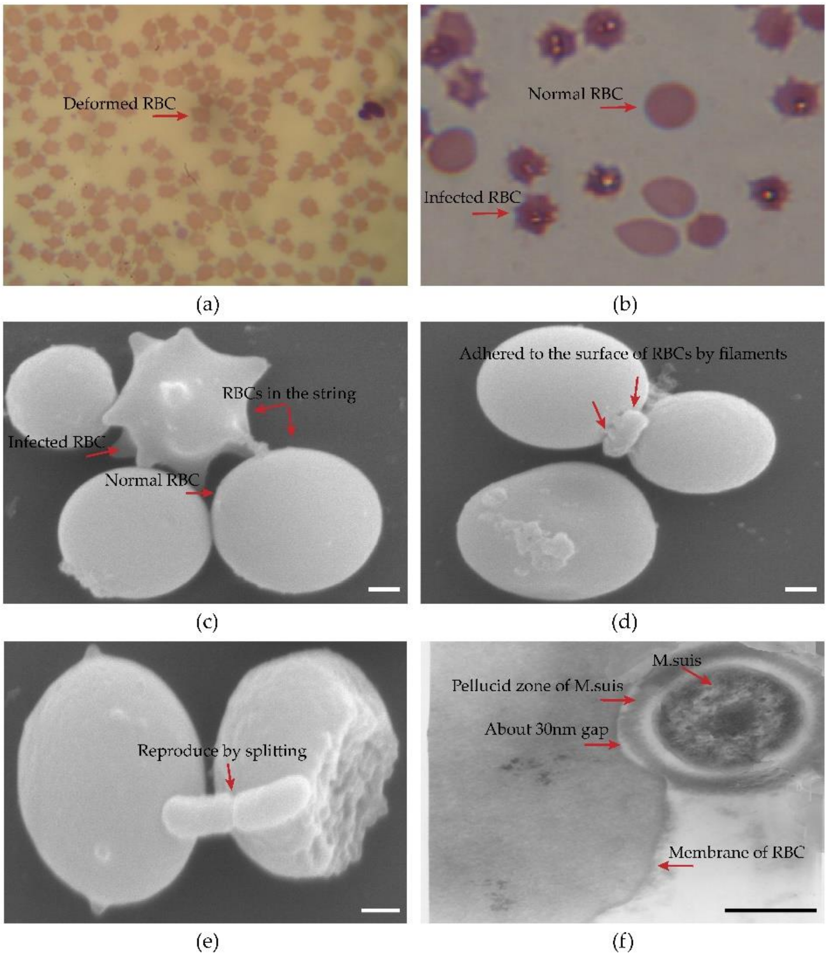

Supplementary Figure S2. The morphological features of high-risk *M.suis* under microscopic state from infected pigs. In Figure S2a and S2b, the fresh blood smears were stained by Wright Giemsa and observed under optical microscope. Figure S2c to S2e were observed under scanning electron microscopy (SEM). Figure S2f was observed under transmission electron microscopy (TEM). Of them, areas of interest were marked by red arrows. (a) Infected red blood cells (RBCs) membrane presented many promontories on the surface; Magnification, ×500. (b) A difference was shown between normal and infected RBCs; Magnification, ×1,000. (c) The normal RBCs were in the string with infected RBCs that were severely deformed and had an irregular shape with many sharp corners; Scale bar = 2 μm. (d) *M.suis* adhered to the surface of RBCs by filaments; Scale bar = 2 μm. (e) The reproduction of mature *M.suis* was observed in a divided state; Scale bar = 1,500 nm. (f) The membrane of RBCs had surface indentation caused by M.suis. About 30 nm gap was found between RBC membrane and *M.suis*. A pellucid zone exited around *M.suis*, full of matrix substance with dark circle areas in deep. The diameter of *M. suis* was about 0.8 μm; Scale bar = 300 nm.

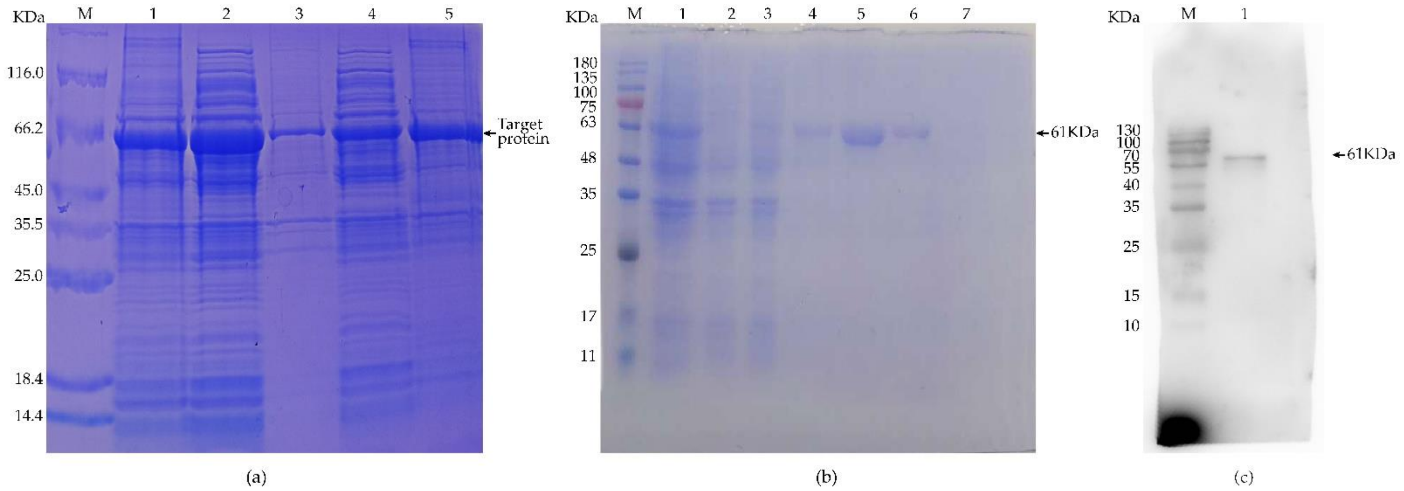

Supplementary Figure S3. Expression, purification of recombinant protein, and full-length blots in parallel experiments. Figure S3a, S3b, and S3c were expressions, purification, and blot of recombinant *Mycoplasma suis* alpha-enolase and kept consistent with Figure 2.

Supplementary Table S1. The results after challenge experients of piglets.

Indicators	Group A	Group B	Group C
High Fever<br>(Yes or No)	Yes	Yes	Yes
Slow weight gain (weekly)<br>(Yes or No)	Yes	Yes	Yes
Anemia<br>(Yes or No)	Yes	Yes	Yes
Find pathogens after challenge<br>(Hour and piglet number)	48h (Three piglets)	24h (Two piglets)<br>48h (One piglet)	24h (Three piglets)
